# Urethral reconstruction using amniotic membrane allograft in hereditary androgen insensitivity syndrome: a case series

**DOI:** 10.1093/jscr/rjad652

**Published:** 2023-12-06

**Authors:** Marah Mansour, Maria Raya, Abd Alrahman Jrdy, Abdoul Majid Sires, Jad Alhaq Wardeh, Almoataz Ballah Alsbekhan, Sabah Faour, Mahmoud Kanas, Mhd Firas Safadi, Khaled Alrebdawi

**Affiliations:** Faculty of Medicine, Tartous University, Tartous 95747, Syrian Arab Republic; Faculty of Medicine, University of Kalamoon, Damascus, Syrian Arab Republic; Faculty of Medicine, Tishreen University, Latakia, Syrian Arab Republic; Faculty of Medicine, University of Aleppo, Aleppo, Syrian Arab Republic; Faculty of Medicine, University of Aleppo, Aleppo, Syrian Arab Republic; Faculty of Medicine, Damascus University, Damascus, Syrian Arab Republic; Faculty of Medicine, University of Aleppo, Aleppo, Syrian Arab Republic; Faculty of Medicine, Yuzuncu Yil University, Van, Turkey; Department of General and Visceral Surgery, Erzgebirgsklinikum Annaberg, Annaberg-Buchholz 09456, Germany; Department of Urological Surgery, Surgical Kidney Hospital, Damascus, Syrian Arab Republic

**Keywords:** androgen insensitivity syndrome, androgen receptor insensitivity syndrome, sexual development, gender transition, amniotic membrane allograft

## Abstract

Partial androgen insensitivity syndrome is a rare X-linked disorder. While most cases are sporadic, familial cases are less frequent. The management of this syndrome follows a multidisciplinary approach involving hormone substitution, psychological counseling, and surgical procedures. We present a case series of three young siblings with familial partial androgen insensitivity syndrome who presented with a female phenotype. All of them were managed with hormonal treatment for 6 months followed by surgical reconstruction. The operative procedure involved phalloplasty and urethroplasty using amniotic membrane transplant, which is considered a novel technique in this group of patients. No intraoperative or postoperative complications were observed and good results were achieved within 2 years of follow-up.

## Introduction

Androgen insensitivity syndrome (AIS) is an X-linked disease caused by a defective androgen receptor (AR) and presents clinically in various phenotypes. The AIS phenotypic spectrum is related to the active ARs and ranges from a complete female phenotype to a male phenotype with undervirilization and/or infertility [1]. The ARs are encoded by the *AR gene*, which is located at *Xq11–Xq12* on the *X-chromosome*. Most AIS cases are sporadic, resulting from new mutations in the *AR gene*, while familial cases are seen in only 10–15% of cases [2]. The clinical manifestations do not differ between sporadic and familial cases, although the latter are usually detected earlier due to the family history [3]. In this article, we present a series of three siblings who were born with female phenotypes and undescended testicles. Two of the patients were subjected to male transformation procedures after hormonal pretreatment with surgical reconstruction using amniotic membrane transplantation. Only a few reports in the literature describe the familial cases of AIS. In addition, this is the first series that reports the use of amniotic membranes for urethral reconstruction in this syndrome.

## Case presentation

### Case 1

**Figure 1:**
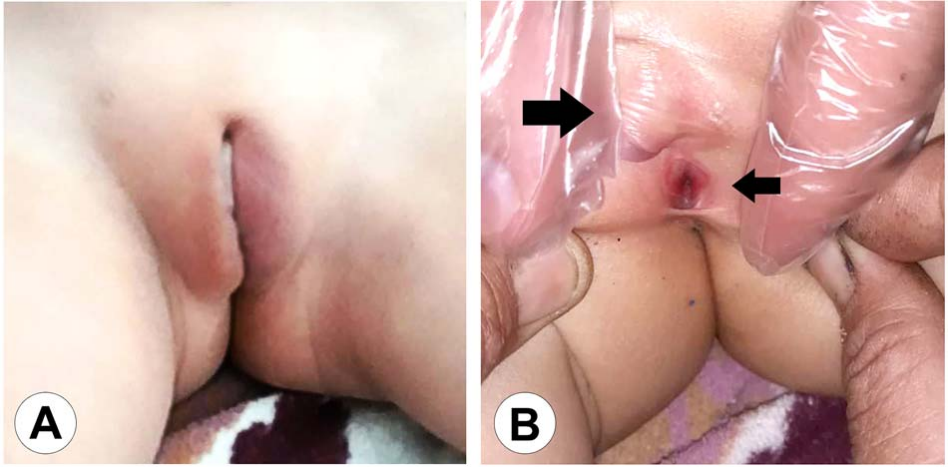
The genital area of Case 1 on presentation. (A) The external genital organs correspond to a feminine appearance with developed labia majora. (B) Retraction of the labia exposes the clitoromegaly (large arrow) and the perineal urethral meatus (small arrow). The examination shows a primitive vestibule with a blind vaginal stump.

**Figure 2:**
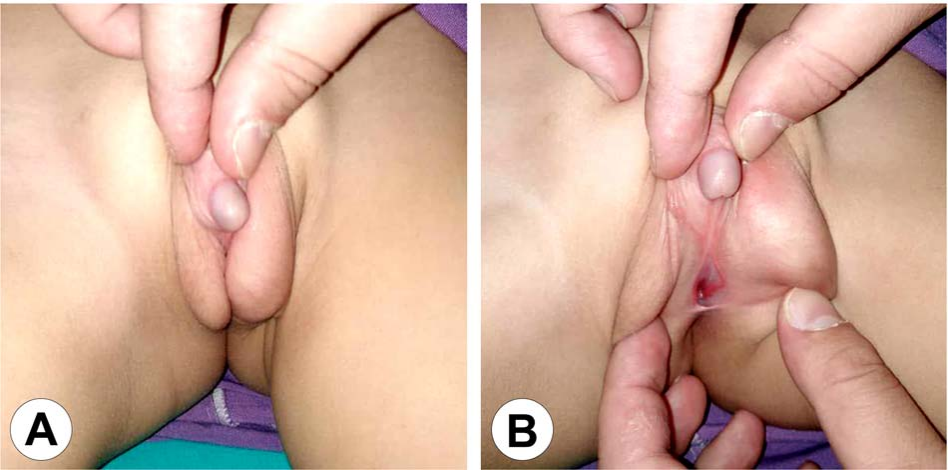
The genital area of Case 1 after 6 months of hormonal therapy. (A) The scrotum shows marked development and the penis can be seen protruding between the scrotal clefts. (B) A perineal hypospadias can now be identified at the root of the scrotum.

**Figure 3:**
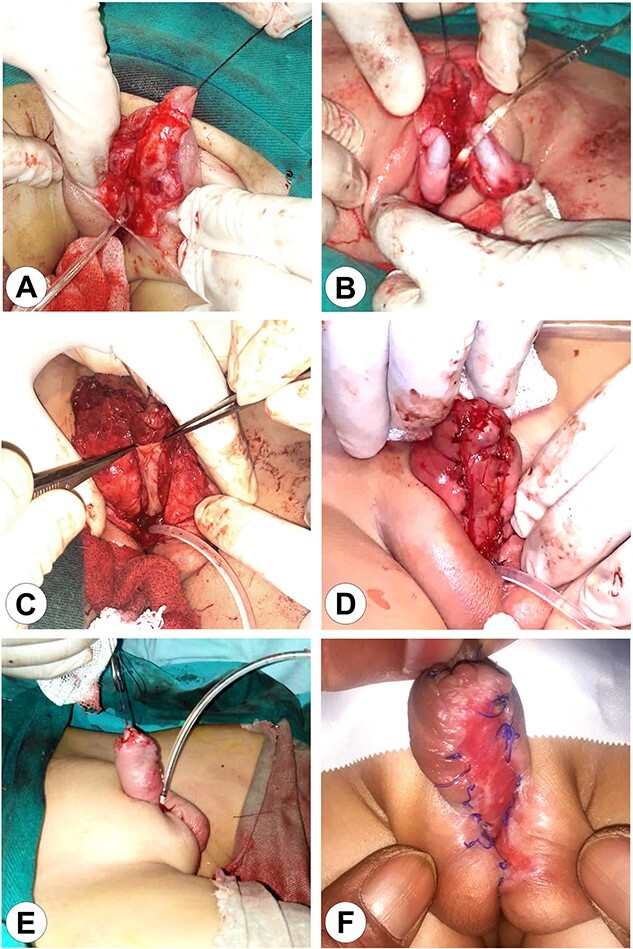
The first stage of the surgical reconstruction. (A) Preparation of the skin flaps with penis release and scrotal reconstruction. (B) Bilateral orchidopexy. (C) Transplantation of an autograft from the patient’s lip on the ventral surface of the penis. (D) Fixation of the implant. (E) The completed reconstruction with a Foley catheter in the perineal urethra. (F) The completed first stage after wound healing.

**Figure 4:**
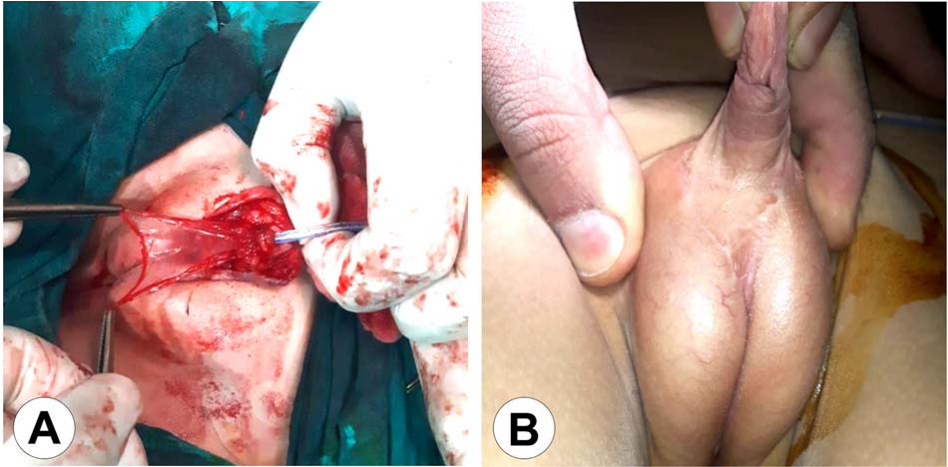
The second stage of the surgical therapy. (A) The urethra was lifted from the scrotum to the glans reinforcing the urethral reconstruction using an amniotic membrane. (B) The final result after wound healing.

A 6-year-old female was admitted to the Urological Surgery Department due to a recently detected swelling in the right inguinal region. No pain, nausea, vomiting, or fever were reported. The patient had no medical or surgical history, or family or genetic disorders. On clinical examination, both the right and the left inguinal areas showed slight swelling. The palpation detected no cough impulse or signs of an inguinal hernia, but rather bilateral palpable soft masses in the inguinal canal. Examination of the external genital organs revealed a clitoromegaly with a primitive vestibule and blind vaginal stump (Fig. 1). An abdominal, pelvic, and inguinal ultrasound revealed bilateral oval-shaped structures between the external inguinal rings and the labia majora, measuring 19 × 13 × 8 mm on the right and 18 × 12 × 7 mm on the left. The structures appeared well-demarcated and homogeneous and were consistent with testes. No uterus or ovaries were detected in the abdomen. The initial diagnosis was directed toward a disorder of sexual development with a phenotypically female appearance. Molecular and cytogenetic karyotyping showed a male karyotype 46 XY, which was performed. The laboratory profile showed normal levels of testosterone, which complies with partial androgen insensitivity syndrome (PAIS). After endocrinological and surgical consultations as well as intensive discussions with the parents, the transformation to the male gender was decided. The therapy was initiated with hormonal treatment using testosterone. Six months later, the penis protruded within the bifid scrotum (Fig. 2) and the patient was ready for surgical reconstruction. The first stage of surgery (phalloplasty) involved constructing a penis and covering it with skin, closing the scrotum, and pulling the testicles down from the groin region (Fig. 3). In the second stage (urethroplasty), the urethra was constructed using an autograft from the patient’s lip. Finally, an amniotic membrane graft was used to support the reconstructed urethra (Fig. 4). The patient was discharged a few days later without any complications. After 2 years of follow-up, the patient was in good physical and psychological condition.

### Case 2

A 5-year-old female, a sibling of the first patient, was admitted to the Urological Surgery Department with the same manifestations. The examination showed a similar phenotypically female appearance. Furthermore, the investigations confirmed a PAIS with a male karyotype. The patient followed the same management strategy with multidisciplinary consultations, hormonal pretreatment, and subsequent two-staged reconstruction after 6 months. The patient showed no complications or psychological sequelae after a follow-up period of 2 years.

### Case 3

**Figure 5:**
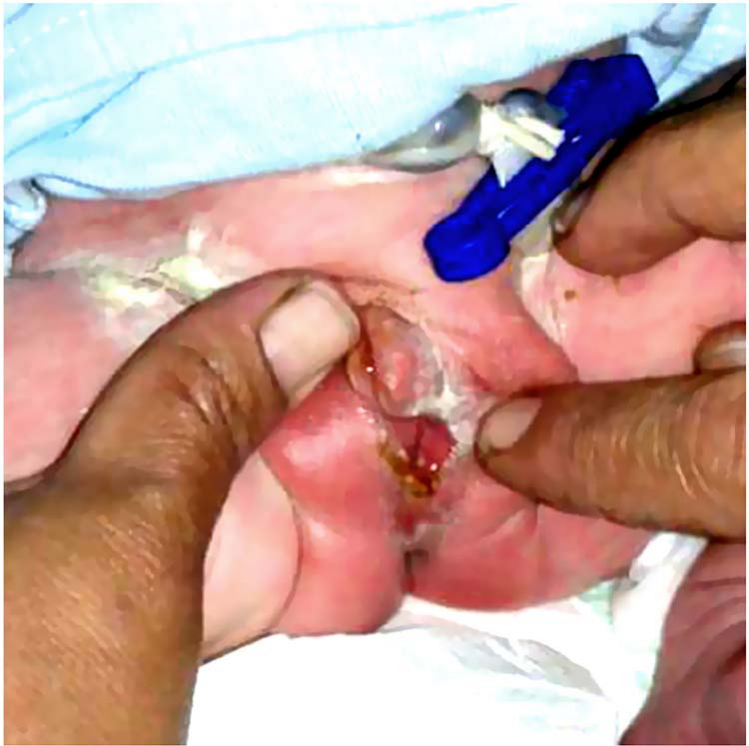
The genital area of a newborn brother of the patients in this series (Case 3) shows the same anomaly with a female pattern and clitoromegaly.

During the treatment of the previously mentioned two patients, their mother became pregnant again. After delivery, the one-day-old newborn was brought for evaluation due to the parent’s concerns. Physical examination revealed female external genitalia (Fig. 5). Furthermore, the workup confirmed the presence of PAIS in the third child. The patient underwent the same treatment strategy with hormonal therapy followed by surgical reconstruction. Postoperatively, the patient was in good condition and showed no complications.

## Discussion

AIS is an X-linked genetic disease, which is considered the most common cause of sex development disorders in 46 XY individuals [4]. The estimated prevalence of AIS varies widely in the literature and ranges from 1:20400 to 1:99100 [5]. The syndrome represents a spectrum of defects in androgen action and can be subdivided into three broad phenotypes: complete androgen insensitivity syndrome (CAIS) with typical female external genitalia; PAIS with predominantly female, predominantly male, or ambiguous external genitalia; and mild androgen insensitivity syndrome (MAIS) with typical male external genitalia [6]. AIS arises from loss-of-function mutations in the coding sequence of the ARs [7]. The *AR gene* is located on the *XqI1.12 chromosome* and mediates the androgens’ activity in expressing the male phenotype [8]. Mutations of the ligand-binding region make the AR unable to bind to the ligand completely, resulting in androgen insensitivity [9]. Most cases of this syndrome are sporadic, resulting from de novo mutations of the *AR gene*. On the other hand, familial cases are less frequent and result from inherited mutations, which may affect many members of the family, as in this case series. Familial AIS was reported in ~15 cases in the medical literature. All were related to mutations in the *AR gene*. McPhaul *et al.* analyzed the relevant coding segment in a family with a qualitative defect in the AR and confirmed the presence of the same genetic alterations in the family members [10]. Chu *et al.* reported 14 affected males in one large Chinese family and identified an *Arg (840) Cys* substitution in the *AR gene* [11]. Kota *et al.* identified four affected family members in India with a *C 2754* to *T* transition in exon 6 of the *AR gene* [8]. In another Brazilian family, five members were shown to be hemizygous for the change of the c.3015C&gt;T nucleotide in *exon 7* of the *AR gene* [2]. So far, the AR Mutations Database (http://androgendb.mcgill.ca/) includes >600 mutations involved in AIS [12]. Approximately 90% of molecular defects in the *AR gene* are single base mutations, mostly missense mutations. Possible clinical indications of AIS can be possibly sustained by a positive *X-linked* family history [13]. Therefore, the molecular analysis of familial AIS is very informative for affected individuals and other family members [14]. Although not tested in the family described in this series due to the lack of resources, we assume that the mother of the three affected children is a carrier of a heterozygous *X-linked* mutation of the *AR gene* that was passed to her male offspring. Patients with PAIS exhibit a broad spectrum of manifestations according to the extent of the *AR gene* mutation [2, 15], which results in a range of abnormalities from female-like to male-appearing external genitalia [4, 16]. Internal genitalia is typically absent due to anti-Mullerian hormone (AMH) action, although the distal part of the vagina may be present since it is not influenced by this hormone [4, 17]. In addition, PAIS can present with gynecomastia, cryptorchidism, and sparse or absent pubic and axillary hair. Familial cases do not differ from sporadic cases regarding manifestations, although the diagnosis may be made at a younger age, as in the third patient in this series [3]. Some reported complications include testis tumors, reduced bone mineral density, infertility, and gynecomastia [17, 18]. The diagnosis of AIS relies on clinical manifestations, laboratory results, and gene analysis. It should be suspected in neonates with female external genitalia and a 46 XY karyotype or high AMH or testosterone levels, female children with inguinal hernia and palpable gonads, and at puberty in females with primary amenorrhea or males with gynecomastia or infertility. Imaging studies can reveal vaginal shortening, undescended testes, and absent uterus [6, 19]. The hormonal analysis shows normal to slightly elevated testosterone, luteinizing hormone, and follicular stimulating hormone. *AR gene* mutations are found in 95% of CAIS patients, but less frequently with PAIS (28–50%) [4, 15, 16]. There are no established criteria for PAIS management, but gender assignment is considered a crucial first step [9, 20]. The patient’s sex is assigned based on the virilization rate of the external genitalia at birth[21]. Nevertheless, a multitude of factors should be considered before choosing the sex of rearing such as the degree of labio-scrotal fusion, the opening site of the urethra, the location of the testes, and the phallus size [15, 20]. Generally, if the patient was raised as a male, they should be allowed to stay a male while performing the necessary genital repair. If the patient was raised as female, early bilateral gonadectomy is recommended to avoid the risk of testicular malignant transformation. In this case, estrogen replacement therapy will be at puberty. [4] As described in all three cases in this series, a multidisciplinary approach by endocrinology, surgery, and psychiatry is highly recommended to determine gender identity and guide the patients or their families to the appropriate therapeutic decisions [22]. Surgical reconstruction can be performed after a hormonal pretreatment with testosterone [3]. After orchidopexy, the reconstruction is usually performed in two stages: phalloplasty and urethroplasty, as described in this case series. The amount of erectile tissue and the extent of hypospadias present important determinants of the success of the procedure [23]. Some complications that may occur after masculinizing genitoplasty may include curvature and chordee of the penis, flap necrosis, or hypospadias, which may require additional corrective procedures. In addition, some patients may develop gynecomastia after puberty, which may require mammoplasty [23]. The first clinical report about using the human amniotic membrane allograft to repair an anterior urethral defect was published in 2020 [24]. Previously, amniotic membranes were used in hypospadias repair, complex reconstruction in Peyronie’s disease, and posterior urethroplasty after pelvic irradiation [25]. Numerous studies showed that amniotic membrane allografts have biological advantages such as inflammation modulation, scar formation reduction, barrier properties, wound healing enhancement, pain reduction, and immunological and antibacterial effects [25]. In addition, amniotic membrane grafts are an appropriate choice for anterior urethral construction, since they are applicable, inexpensive, and easy to apply. They provide satisfying cosmetic results and may reduce the formation of urethral fistulas [24]. Amniotic membrane allograft was successfully used for reconstruction in our cases 1 and 2 with excellent results.

## Conclusion

Familial PAIS is an inherited disorder that makes the patient’s phenotype different from his genotype. Appropriate management requires multidisciplinary collaboration and the use of hormonal and surgical therapy. In this article, we presented a case series of three siblings with this syndrome and we reviewed the literature for uncommon familial presentations. The three patients underwent male transformation procedures using amniotic membrane allograft as a novel and advantageous way of covering the urethral defect. More studies are needed to evaluate the applicability of amniotic allografts in urethral reconstruction.

## Data Availability

Not applicable. All data (of the patient) generated during this study are included in this published article and its supplementary information files.
